# Oxidative Stress and Free-Radical Oxidation in BCG Granulomatosis Development

**DOI:** 10.1155/2013/452546

**Published:** 2013-04-23

**Authors:** Elena Menshchikova, Nikolay Zenkov, Victor Tkachev, Oksana Potapova, Liliya Cherdantseva, Vyacheslav Shkurupiy

**Affiliations:** ^1^Research Center of Clinical and Experimental Medicine, USA; ^2^University of Michigan School of Medicine, USA; ^3^Novosibirsk State Medical University, Russia

## Abstract

*Background*. Little is known about the role of free-radical and oxidative stress signaling in granuloma maturation and resolution. We aimed to study the activity of free-radical oxidation processes in the dynamics of BCG-induced generalized granulomatosis in mice. *Methods*. Chronic granulomatous inflammation was induced in male BALB/c mice by intravenously injecting the BCG vaccine, and the production of oxidative stress (activity of free-radical oxidation processes) and histological changes in the lungs, liver, and peritoneal exudate were measured 3, 30, 60, and 90 days after infection. *Results*. The tuberculous granuloma numerical density and diameter continuously increased from day 30 to day 90, and the macrophage content within the granulomas progressively diminished with a concomitant elevation in the number of epithelioid cells. The activity of the free-radical oxidation processes in the liver (i.e., the intensity of the homogenate chemiluminescence) reached a maximum at postinfection day 60 and subsequently began to decrease. The peak generation of reactive oxygen species by phagocytes in the peritoneal exudate (measured using flow cytometry) was also shifted in time and fell on day 30. *Conclusions*. The rise in the steady-state concentration of H_2_O_2_ in the liver of mice with BCG-induced granulomatosis is not related to local H_2_O_2_ production by phagocytes, and a decrease in the severity of generalized inflammation precedes the resolution of local inflammation.

## 1. Introduction

The generation of inflammatory granulomas that result from the proliferation and transformation of phagocytic cells is a hallmark of many infectious (e.g., tuberculosis (TB) and tularemia) and noninfectious (e.g., silicosis, asbestosis, and granulomatous hepatitis) diseases (more than 70 disease entities) [1, 2]. When it is necessary to isolate foreign objects, including microorganisms, granulomatous inflammation is generally believed to appear; these objects cannot be removed by the normal process of phagocytosis (with subsequent degradation). However, researchers face several complicated problems when studying this phenomenon. For instance, the appearance of idiopathic granulomas with an inducer of unknown nature is a distinguishing feature of sarcoidosis, Wegener's disease, and several other diseases [2, 3]. Another problem is the almost complete lack of effective tools and techniques to influence granulomatous processes; anything offered by modern medicine is essential either the therapeutic removal of an inducer (for infectious granulomas) or the surgical removal of the affected organ. Another problem is the high unpredictability in the development and resolution of inflammatory granulomas; although these processes depend on inducing factors, they are notably individual. If we consider the problem of granulomogenesis in general, the least studied topic is the participation of reactive oxygen and nitrogen species (RONS) as an evolutionarily ancient mechanism of intra- and intercellular regulation; this regulation orchestrates (by means of redox signaling) cell migration, cooperation, functional activity, life cycle, proliferation, and death [4]. 

A classic example of infectious granulomatosis is TB, the most common infectious disease in the world. At the heart of its development and manifestation is the persistence of the pathogen *Mycobacterium tuberculosis,* mainly in the vacuolar apparatus of macrophages cells that are specialized in antibacterial protection, 90% of which are determined by oxygen-dependent mechanisms [3]. There are many researches that consider both the mechanisms of free-radical antimycobacterial host phagocyte protection and the methods by which *M. tuberculosis* avoids RONS attack [3, 5–7], as well as oxidative stress development. However, up to now, no effective redox-dependent methods to treat TB or control an *M. tuberculosis *infection have been proposed. The high incidence of TB in people with NAD(P)H oxidase genetic defects [7] and the prospects for targeted delivery of nitric oxide using inhalable microparticles containing NO donors [8] indicate the need for better understanding of the role of free-radical processes involving RONS in *M. tuberculosis *persistence during the dynamics of TB granulomatosis.

For the first phase of studying the roles of oxidative stress and reactive oxygen species (ROS) as effectors and regulators of granulomatous inflammation, we attempted to examine the change in the activity of free-radical oxidation processes during the dynamics of chronic BCG-induced generalized granulomatosis in mice and compared this change with the morphological changes.

## 2. Materials and Methods

### 2.1. Animal Model

The Animal Care Committee of the Research Center of Clinical and Experimental Medicine approved the experimental protocol. Male BALB/c mice (weight: 18–22 g, age: 2 months) were purchased from the Research Institute of Clinical Immunology SB RAMS (Novosibirsk, Russia). The mice were housed in an environment with controlled temperature and controlled light and divided into eight groups (*n* = 5 in each group) as follows: four groups with a model of generalized tuberculous granulomatosis (3, 30, 60, and 90 days after a single injection of 0.5 mL of BCG vaccine (Microgen, Russia) in 1 mL of saline into the tail vein) and four control groups (3, 30, 60, and 90 days after a single injection of 1 mL of saline into the tail vein) [1, 2]. The animals were weighed and sacrificed by cervical dislocation. Peritoneal leukocyte samples were obtained to evaluate the oxidative metabolism of these cells. The livers and lungs were quickly removed, weighed, and processed for histological examination and preparation of liver homogenates. These organs were selected because they are the most often affected in generalized tuberculosis and they also contain the largest compartment of cells of the mononuclear phagocyte system, which form the basis of granulomas.

### 2.2. Histological Examination

Liver and lung fragments were fixed in 10% neutral formalin, dehydrated in ascending alcohol solutions, and embedded in paraffin. The sections (4-5 *μ*m thick) were stained using the hematoxylin/eosin and Van Gieson/Elastin techniques and studied using light microscopy (AxioImager A1, Carl Zeiss, Germany). Specific histochemical staining by the Ziehl-Neelsen stain was used to visualize *Mycobacterium bovis* in the tissues. Using the morphometry method (AxioVision software, rel. 4.8), the numerical density of the granules and their diameters were determined; these parameters were used as the morphological criteria for the tuberculosis activity. This activity is caused by the chemoattractant gradient, which is created by alive mycobacteria (free and persistent in macrophages). The granule size showed the value of the chemoattractant potential [1, 2]. 

### 2.3. Activity of Free-Radical Oxidation Processes

#### 2.3.1. Chemiluminescence (CL)

The livers were rinsed with saline, minced with scissors, and homogenized on ice in a Potter-Elvehjem tissue grinder with 5 vol (w/v) of Hanks' balanced salt solution without phenol red (HBSS) (200 mg/mL). After recording the background CL of the measuring cuvette at 37°C in a chemiluminometer (Photon, Russia) for 2 minutes, 2 mL of liver homogenate was placed in the cuvette and then incubated for 2 minutes, after which the spontaneous CL was measured for 2 minutes. Afterward, 0.1 mL of 100 nM luminol (Serva, Germany) solution was injected, the luminol-amplified CL (LACL) was measured for 2 minutes, 0.1 mL of H_2_O_2_ solution was then added (final concentration 39.5 mM), and the H_2_O_2_-induced luminol-amplified CL (H_2_O_2_-LACL) was measured. The CL intensity was expressed in arbitrary units (1 a.u. =5 impulses/s) with each value representing an average. The averaged background CL intensity of the measuring cuvette was subtracted from the averaged values for the spontaneous and luminol-amplified chemiluminescence.

#### 2.3.2. Flow Cytometry

Peritoneal exudate cells were obtained by peritoneal lavage with cold RPMI 1640 medium (Biolot, Russia) supplemented with 1% (v/v) fetal bovine serum (Biolot), and kept on ice until measurement. To measure the total ROS production, isolated cells were incubated for 15 min in 1 mL of HBSS containing 10 mM 2′,7′-dichlorodihydrofluorescein diacetate (Sigma, USA) or 50 mM dihydroethidium bromide (Sigma). The former is deacylated intracellularly and rapidly oxidized by ROS to yield the highly fluorescent product 2′,7′-dichlorofluorescein (DCF), and oxidation of the latter molecule, which is not fluorescent, in cells by superoxide anion radicals results in the formation of 2-hydroxyethidium (2OH-E), whose fluorescence is in the red. 

We investigated both spontaneous ROS and the ROS stimulated with 100 nM phorbol 12-myristate 13-acetate (PMA, Sigma). Using the FACSCalibur (Becton-Dickinson, USA) flow cytometer, we measured the intensity of the DCF-dependent fluorescence (*λ*_Em_ = 488 nm, *λ*_Ex_ = 520 nm), which is predominantly an indicator of H_2_O_2_ generation by cells, and the 2OH-E fluorescence (*λ*_Em_ = 488 nm, *λ*_Ex_ = 630 nm), which is mainly sensitive to the superoxide anion. The gating of the viable macrophages and granulocytes was based on light scattering (forward and side scatter) properties. The results of the cell fluorescence intensity were normalized to the spontaneous fluorescence in control mice, taken as 100%.

### 2.4. Statistical Analysis

The Kolmogorov-Smirnov test was used to check whether the variables were normally distributed. For variables with a normal distribution, the parametric *t*-test was used for two independent samples, and the data are represented as the mean ± SEM. Variables that were not distributed normally were evaluated using the Mann-Whitney nonparametric test, and the data are represented as the median and the lower (*Q*_1_) and upper (*Q*_3_) quartiles. The relationships between the variables were assessed by Spearman's rank correlation coefficient (*r*). *P* values less than 0.05 were considered significant. 

## 3. Results

### 3.1. Histological Examination

Histological examination revealed that 30 days after infection the mice developed disseminated tuberculous inflammation, which was manifested morphologically by BCG granuloma formation in the internal organs and visceral membranes. *M. bovis* bacteria were detected in the foci of the granulomatous inflammation. However, necrotic changes in the granulomas of the lungs and liver of the mice were not found in any experimental group. This result was most likely caused by the weakened virulence of *M. bovis* in BCG (used to vaccinate the animals), and, therefore, by the decrease in the chemotactic capacity and the direct effect of the mycobacterial cell wall lipid components on granuloma cells [1, 2].

**Figure 1:**
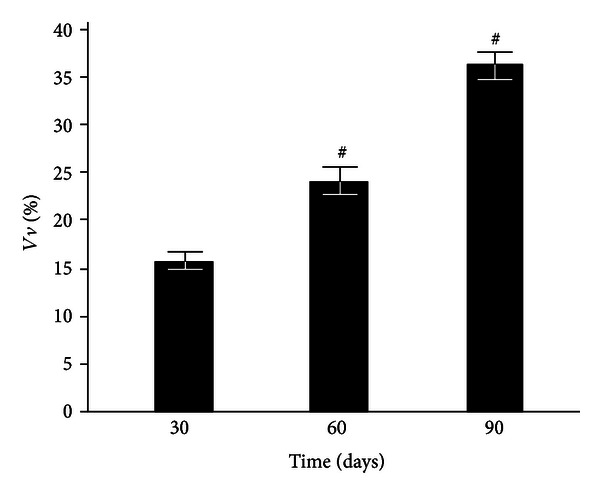
Volume density of mononuclear infiltrates in the lung interstitium of control and BCG-infected mice. The results represent the mean ± SEM. ^#^The value differs significantly from the value for the previous observation period (*P* < 0.05).

The numerical density of granulomas in the liver and lungs increased by 2.7 and 1.5 times, respectively, throughout the 30–90th days of infection, and simultaneously, the granuloma diameter increased by 24.6% in the liver and 43.1% in the lungs (Table 1). The study of the granuloma cellular composition revealed that the macrophage, neutrophil, and lymphocyte numbers consistently declined (the lung neutrophil count did not change), but the numbers of epithelioid cells and fibroblast increased. This finding indicates a stable course of tuberculosis with a tendency toward progression and no propensity toward a spontaneous cure. The numerical density and diameter of the granulomas in the lungs were higher than those in the liver at all stages of observation (Table 1). The number of epithelioid cells in the liver and lung granulomas did not differ significantly at day 30 after infection and increased by 3.5 and 2.2 times, respectively, at day 90. Simultaneously, an elevation of lung mononuclear infiltrates in the interstitium was observed; this volume density was enhanced 2.3-fold from the 30th to the 90th day (Figure 1).

Such differences in the morphogenesis of the tuberculous granulomatous inflammation in the liver and lungs of experimental animals can be related to the structural features of alveolar macrophages, which contain a large number of lysosome-like structures in the cytoplasm, and the close topographical interrelation of all alveolus wall components, both among themselves and with blood elements [9]. In addition, because pulmonary macrophages function in an aerobic environment with increased oxygen tension, the major bactericidal mechanism in the lungs is free radicals [9, 10]. Thus, excess RONS generation leads to both destabilization of cell membranes and lung tissue damage. In turn, degradation products can attract new populations of macrophages and T cells to the lesion area. Thus, the granuloma diameter increases via an expansion of the peripheral zone, which is represented by mononuclear cells (macrophages and lymphocytes), and via diffuse infiltrative alterations of the interstitium similar to mononuclear alveolitis [9].

### 3.2. Activity of Free-Radical Oxidation Processes

#### 3.2.1. Chemiluminescence (CL)

**Figure 2:**
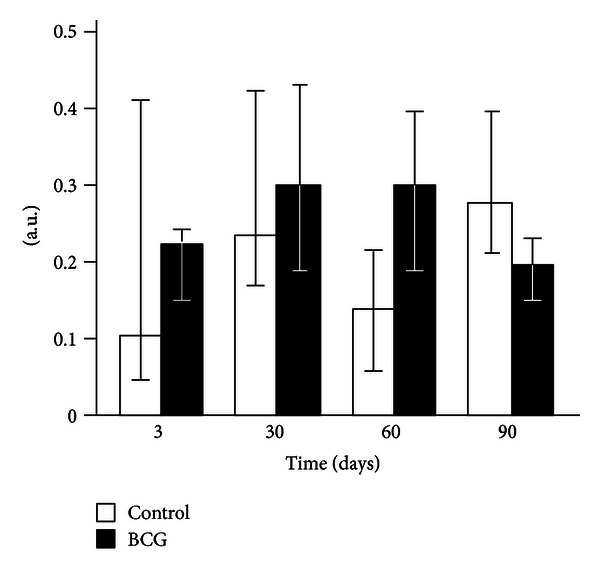
Spontaneous chemiluminescence of liver homogenates of control and BCG-infected mice. The bar represents the median value, and the error bar indicates the lower and upper quartiles.

**Figure 3:**
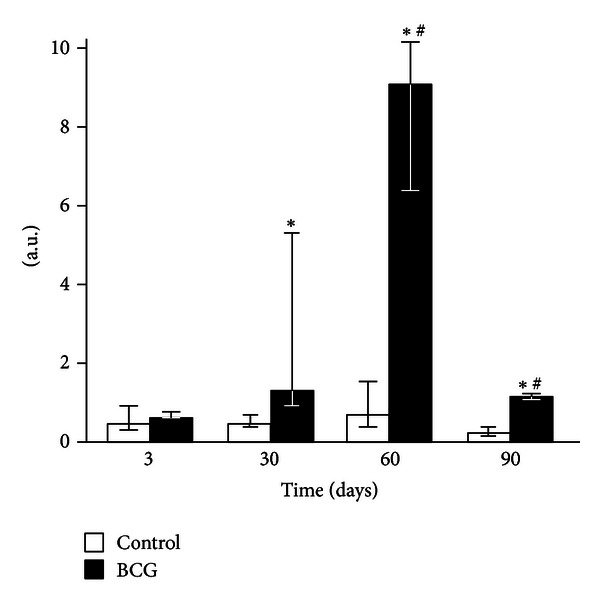
Luminol-amplified chemiluminescence of liver homogenates of control and BCG-infected mice. The bar represents the median value, and the error bar indicates the lower and upper quartiles. ^#^The value differs significantly from the value for the previous observation period (*P* < 0.05). *Significant difference between the BCG and control groups (*P* < 0.05).

**Figure 4:**
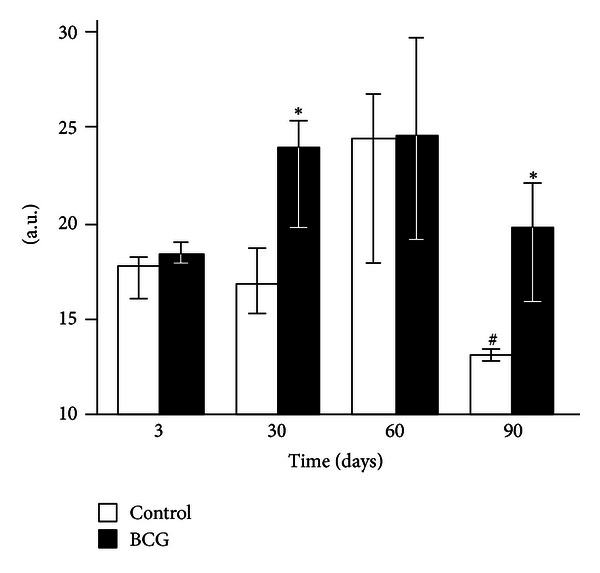
Luminol-amplified H_2_O_2_-induced chemiluminescence of liver homogenates of control and BCG-infected mice. The bar represents the median value, and the error bar indicates the lower and upper quartiles. ^#^The value differs significantly from the value for the previous observation period (*P* < 0.05). *Significant difference between the BCG and control groups (*P* < 0.05).

None of the liver homogenate CL parameters changed 3 days after the BCG administration to mice (Figures 2–4). After 30 days of infection, the spontaneous CL did not differ significantly from the control values, but when luminol was introduced into a registration system, we observed a significant increase in the light intensity in the BCG-injected group, which was even more pronounced for the CL induction by hydrogen peroxide (Figures 2–4). The LACL value enhancement was even more pronounced 60 days after-infection and was not only 8.7 times higher than that in control but also significantly greater than the LACL intensity of the liver homogenates of mice 30 days after the BCG vaccine injection (Figure 3). The H_2_O_2_-LACL values in the experimental and control groups were similar for the same observation period (Figure 4). After 90 days of the experiment, the control and experimental mice differed in both the LACL and the H_2_O_2_-LACL intensities (Figures 3 and 4), and the latter was paradoxically low in the control group.

When analyzing the relationships between various CL parameters and the body and organ weights, interesting patterns were revealed. For instance, the body weight of the control animals was correlated to a significant extent with the H_2_O_2_-LACL and the liver and lung weights. After intravenous administration of the BCG vaccine, the liver weight was also positively correlated with the homogenate LACL and the lung weight. Furthermore, there was a negative correlation between the lung and H_2_O_2_-LACL, whereas the inverse relationship between H_2_O_2_-LACL and body weight was no longer relevant (Table 2). 

#### 3.2.2. Flow Cytometry

**Figure 5:**
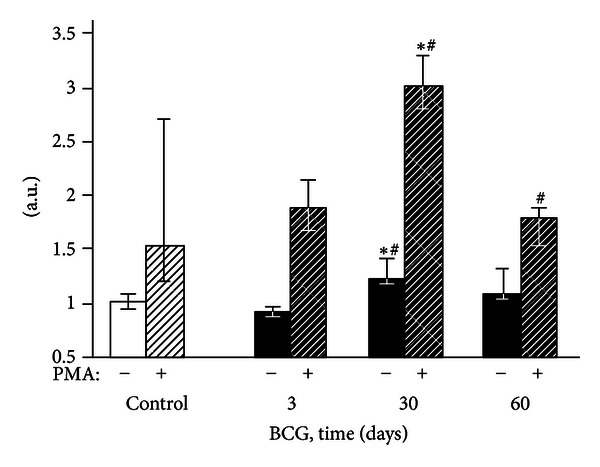
DCF-dependent fluorescence of exudate polymorphonuclear neutrophils of control and BCG-infected mice. The bar represents the median value, and the error bar indicates the lower and upper quartiles. ^#^The value differs significantly from the value for the previous observation period (*P* < 0.05). *Significant difference between the BCG and control groups (*P* < 0.05).

**Figure 6:**
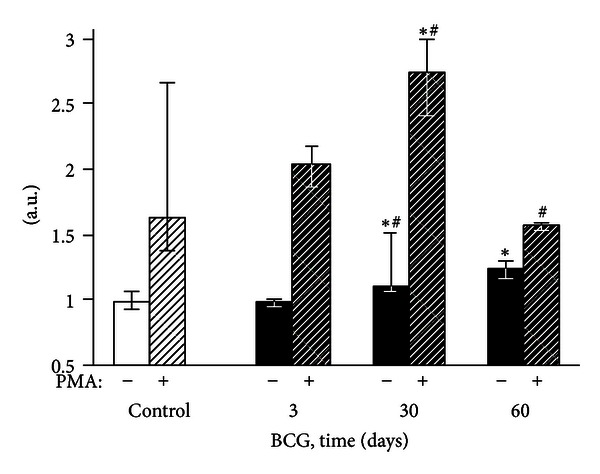
DCF-dependent fluorescence of exudate macrophages of control and BCG-infected mice. The bar represents the median value, and the error bar indicates the lower and upper quartiles. ^#^The value differs significantly from the value for the previous observation period (*P* < 0.05). *Significant difference between the BCG and control groups (*P* < 0.05).

**Figure 7:**
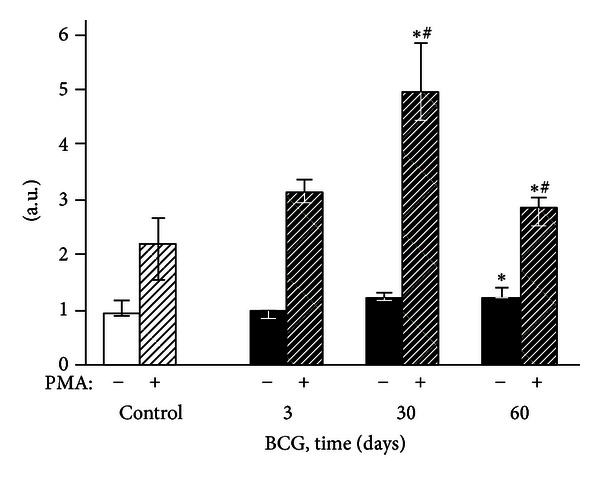
2OH-E-dependent fluorescence of exudate polymorphonuclear neutrophils of control and BCG-infected mice. The bar represents the median value, and the error bar indicates the lower and upper quartiles. ^#^The value differs significantly from the value for the previous observation period (*P* < 0.05). *Significant difference between the BCG and control groups (*P* < 0.05).

**Figure 8:**
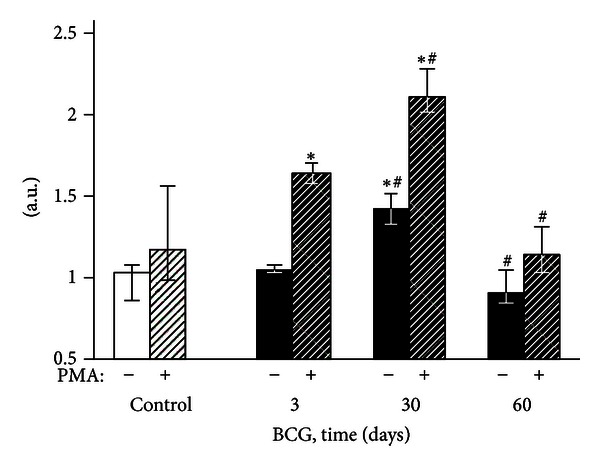
2OH-E-dependent fluorescence of exudate macrophages of control and BCG-infected mice. The bar represents the median value, and the error bar indicates the lower and upper quartiles. ^#^The value differs significantly from the value for the previous observation period (*P* < 0.05). *Significant difference between the BCG and control groups (*P* < 0.05).

Three days after the intravenous BCG vaccine injection, the fluorescence intensity of the peritoneal exudate cells did not differ from the control group values (Figures 5–8). After 30 postinfection days, the spontaneous and PMA-stimulated ROS generation by the exudate granulocytes increased, indicating their metabolic activation (Figures 5 and 7). Moreover, during the same period, both activation (increase of the spontaneous DCF fluorescence intensity) and priming (enhancement of phorbol ester-stimulated DCF fluorescence) of peritoneal macrophages were revealed (Figures 6 and 8). The intensity of H_2_O_2_ and O_2_^−^ generation by the phagocytes in the mouse peritoneal cavities decreased significantly 60 days after the intravenous BCG vaccine injection, mainly returning to the original values. However, the 2OH-ET-dependent granulocyte fluorescence (both spontaneous and after induction of respiratory burst by PMA) and the unstimulated DCF-dependent macrophage fluorescence remained somewhat elevated (Figures 5–8).

## 4. Discussion

Currently, the study of granulomatous inflammation focuses on cytokine regulation despite the nosological identification of hereditary chronic granulomatous disease, which is based on a variety of defects in the membrane NAD(P)H oxidase complex, resulting in phagocytic cells that cannot generate superoxide anion radicals [11]. This fact is interpreted to indicate that ROS interferes with granuloma formation, and the genesis of the latter is mainly due to decreased phagocyte microbicidal activity [12]. However, this interpretation appears superficial because intercellular redox regulation occurs via neutral oxidative stress effectors, primarily nitric oxide (NO^•^) and hydrogen peroxide (H_2_O_2_). Superoxide anion radicals are the most effective scavengers of nitric oxide radicals; thus cells with NAD(P)H oxidase defects are characterized by more active NO-mediated cell-cell communication, which may contribute to granuloma formation.

NO participation in the formation of both infectious and noninfectious granulomas in humans and animals has long been known [13]. The main producer of NO radicals in macrophages and within a granuloma is inducible NO synthase, whose expression is controlled by the transcription factor NF-*κ*B and increases in response to endotoxin, proinflammatory cytokines, and other factors. The main sources of NO^•^ in granulomas are macrophages and giant Pirogov-Langhans cells and, to a lesser extent, epithelioid cells [14]. NO radicals are believed to be a major factor that kills mycobacteria and limits the growth of pathogens in tuberculosis [3]. Tumor necrosis factor *α* and interferon *γ* participate in the host defense against mycobacteria, also by increasing NO production [15]. The inducible NO synthase content in the granulomas of BCG-infected cattle increases, reaching a maximum at day 42 (10 times more than on the 15th day), and then decreases after fibrosis development [16]. Some authors claim that NO synthesis by macrophages determines granuloma organization and development [17]. The molecule serves as a chemoattractant and regulates the differentiation and activation of epithelioid cells [18]. In turn, the expression of inducible NO synthase by granuloma cells is regulated by the NRAMP1 protein [14]. One of the functions of NRAMP1 is to transport bivalent metals (including Fe^2+^ ions) into the phagosome. In the presence of hydrogen peroxide, this transport results in the generation of the hydroxyl radical, ^•^OH, which is fatal for mycobacteria. 

The participation of hydrogen peroxide in the formation and development of granulomatous inflammation has been studied to a lesser extent; however, investigators research not only the cytotoxic potential of H_2_O_2_ as an oxidative stress mediator but also the regulatory potential of the molecule. The H_2_O_2_/eosinophil peroxidase system has been shown to be directly involved in the destruction of *Schistosoma mansoni* eggs in a granuloma [19]. Furthermore, the microbicidal activity of guinea pig alveolar macrophages toward various strains of *M. tuberculosis* and *M. bovis* was not related to the intensity of the respiratory burst and H_2_O_2_ generation, although the efficiency of phagocytosis of different bacterial strains was inversely correlated with their virulence [20]. Both the administration of the compounds that inhibit hydrogen peroxide and neutrophil depletion with a specific antiserum reduced the formation of noninfectious granulomas in a dose-dependent manner [21, 22], particularly when chemokine expression on the surface of endothelial cells (decrease of monocyte chemotactic protein-1 expression) was inhibited [22].

Our study shows that BCG granulomatosis in the liver is not accompanied by a change in free-radical lipid peroxidation activity in the whole organ. This activity was measured by the intensity of spontaneous CL in the liver homogenate, which directly depends on the steady-state concentration of lipid alkoxyl and peroxyl radicals [3, 23] (Figure 2). The activities of free-radical oxidation processes and oxidative stress, measured by the luminol-dependent CL of the homogenates, increased simultaneously with a peak at day 60 after infection (Figure 3). This increase, along with the appearance of a correlation between the liver weight and the intensity of the liver homogenate LACL in BCG-infected animals (Table 2, the lower left part), suggests an increase in the steady-state concentration of H_2_O_2_ in the liver of infected animals. We believe that this H_2_O_2_ increase occurs mainly in the granulomas, although we do not rule out the contribution of adjacent hepatocytes. Abdallahi et al. [24] also revealed that the maturation of *Schistosoma mansoni*-induced granulomas in mouse livers is accompanied by a gradual increase in liver H_2_O_2_ generation. Moreover, the steady-state concentration of hydrogen peroxide also increases outside of the granuloma in neighboring hepatocytes (although to a lesser extent). 

The intensity of the H_2_O_2_-induced luminol-dependent chemiluminescence of biological substrates directly depends on their oxidizability [3]. The fact that on day 60 (in contrast to postinfection days 30 and 90) the addition of exogenous hydrogen peroxide to the liver homogenates did not result in an increase of the luminol-amplified chemiluminescence over the control values (Figure 4) suggests that the H_2_O_2_ production reached maximal values at this time point.

The absence of peritoneal granulocyte activation at the early stages of the experiment (day 3), both spontaneous and PMA-induced (Figures 5 and 7), is consistent with the data indicating that polymorphonuclear leukocytes do not directly capture BCG in the liver (in contrast to Kupffer cells and newly recruited monocytes/macrophages) [25]. In addition, during this period, granulomas are still not formed and are present as an immature small infiltrate. However, granulocytes are involved in the liver and lungs as cells that affect inflammation in mature granulomas (30 days) [1], and this involvement is followed by the activation of oxygen-dependent metabolism. The decrease of ROS generation by peritoneal exudate neutrophils and macrophages begins earlier (at day 60, Figures 5–8) than the decline of activity of free-radical oxidation in the liver (Figure 2). This timing indicates that the mitigation of the severity of generalized inflammation precedes the resolution of local inflammation. We consider the resolution of inflammation as the transition into the production phase, the prevalence of fibrosis over phagocyte migration in the area, and the resulting oxygen-mediated degradation. After postinfection day 60 in the granulomas, the percentage of neutrophils and macrophages, the most active RONS producers, also consistently decreases. This decrease occurs despite the successive enhancement of the number and diameter of granulomas in the liver and lung and the volume density of mononuclear infiltrates in the lungs; these enhancements are mainly due to the increase in the number of epithelioid cells (Table 1 and Figure 1). Thus, it is not clear whether the observed increase in the steady-state concentration of hydrogen peroxide in the liver of infected animals, especially 60 days after BCG vaccination, is due to the direct generation of H_2_O_2_ by phagocytes.

## 5. Conclusions

The study of oxidative stress (produced by the enhancement of endogenous free-radical oxidation processes) and of morphological changes during BCG vaccine-induced chronic inflammation allowed us to conclude the following. In the lungs and livers of mice up to postinfection day 90, the numerical density and diameter of the granulomas sequentially increase mainly due to the enhancement of the epithelioid cell number, whereas the number of phagocytic cells reduces. In contrast, the activity of free-radical oxidation processes in the liver and peritoneal exudate enhances at days 60 and 30 after BCG vaccine injection, respectively, and gradually decreases thereafter. Thus, the rise in the steady-state H_2_O_2_ concentration in the liver of infected animals is not related to its local production by phagocytes, and a decrease in the severity of generalized inflammation precedes the resolution of local inflammation. The data obtained on the uncoupling of H_2_O_2_- and progenitor O_2_^−^-related processes on the systemic (phagocytes in the peritoneal cavity) and local (liver homogenates) levels at different stages of BCG-induced granulomatosis appear to indicate a possible role of hydrogen peroxide in intercellular communication during organization, maturation, and “dissociation” of granulomas in the dynamics of the process.

## Figures and Tables

**Table 1:** Numerical density and cellular composition of BCG granulomas in the liver and lungs of BCG-infected mice.

Parameter	Liver			Lungs		
	Time after infection (days)					
	30	60	90	30	60	90
Numerical density of granulomas (Nai), 3.64 × 10^5^ *μ*m^2^	3.08 ± 0.26	7.84 ± 0.64^#^	8.36 ± 0.54	7.40 ± 0.57	8.24 ± 0.51	11.24 ± 0.46^#^
Diameter of granulomas, *μ*m	47.25 ± 1.26	53.18 ± 1.54^#^	58.92 ± 1.32^#^	49.69 ± 1.24	68.71 ± 0.34^#^	71.12 ± 1.82
Macrophage number, %^$^	65.12 ± 0.69	33.85 ± 0.87^#^	14.72 ± 1.33^#^	47.94 ± 0.53	27.11 ± 0.86^#^	23.13 ± 0.59^#^
Epithelioid cell number, %^$^	22.56 ± 0.87	57.05 ± 0.89^#^	77.35 ± 1.42^#^	26.37 ± 0.23	52.21 ± 1.04^#^	58.21 ± 0.23^#^
Neutrophil number, %^$^	2.47 ± 0.18	0.69 ± 0.14^#^	0.21 ± 0.09^#^	1.83 ± 0.23	2.53 ± 0.49	2.09 ± 0.42
Lymphocyte number, %^$^	7.51 ± 0.30	5.47 ± 0.20^#^	1.64 ± 0.27^#^	12.48 ± 1.21	7.19 ± 0.36^#^	3.92 ± 0.31^#^
Fibroblast number, %^$^	2.34 ± 0.20	2.94 ± 0.18^#^	6.08 ± 0.24^#^	11.38 ± 0.31	10.96 ± 0.36	12.65 ± 0.81^#^

The results represent the mean ± SEM.

^$^100%: the total number of cells in granuloma. ^#^The value significantly differs from the value for the previous observation period (*P* < 0.05).

**Table 2:** Relationship (*r*) between body and organ weights and CL intensity of the liver homogenates of control and BCG-infected mice for the entire period of observation.

	Spontaneous CL	LACL	H_2_O_2_-LACL	Body weight	Liver weight	Lung weight
Spontaneous CL		*0.12 *	*−0.36 *	*−0.18 *	*0.25 *	*−0.06 *
LACL	**0.07**		*0.22 *	*−0.19 *	*0.32 *	*−0.27 *
H_2_O_2_-LACL	**0.06**	**0.46**		−0.62 *	*0.09 *	*−0.44 *
Body weight	**0.24**	**0.22**	**−0.08**		0.51 *	0.65 *
Liver weight	**−0.06**	0.62*	**−0.07**	0.67*		*0.27 *
Lung weight	**−0.09**	**0.12**	−0.57*	0.49*	0.63*	

Upper right part of table: control (in italics); lower left: BCG injection (in bold). *Significant *r* values.
